# Characterizing and Predicting Submovements during Human Three-Dimensional Arm Reaches

**DOI:** 10.1371/journal.pone.0103387

**Published:** 2014-07-24

**Authors:** James Y. Liao, Robert F. Kirsch

**Affiliations:** 1 Department of Biomedical Engineering, Case Western Reserve University, Cleveland, Ohio, United States of America; 2 Cleveland Functional Electrical Stimulation Center, Cleveland, Ohio, United States of America; VU University Amsterdam, Netherlands

## Abstract

We have demonstrated that 3D target-oriented human arm reaches can be represented as linear combinations of discrete submovements, where the submovements are a set of minimum-jerk basis functions for the reaches. We have also demonstrated the ability of deterministic feed-forward Artificial Neural Networks (ANNs) to predict the parameters of the submovements. ANNs were trained using kinematic data obtained experimentally from five human participants making target-directed movements that were decomposed offline into minimum-jerk submovements using an optimization algorithm. Under cross-validation, the ANNs were able to accurately predict the parameters (initiation-time, amplitude, and duration) of the individual submovements. We also demonstrated that the ANNs can together form a closed-loop model of human reaching capable of predicting 3D trajectories with VAF >95.9% and RMSE ≤4.32 cm relative to the actual recorded trajectories. This closed-loop model is a step towards a practical arm trajectory generator based on submovements, and should be useful for the development of future arm prosthetic devices that are controlled by brain computer interfaces or other user interfaces.

## Introduction

We are interested in understanding the process by which people accurately reach with their arms to objects in the environment. Such movements are typically made smoothly and accurately to targets throughout the reachable volume of the arm despite obstacles that must be avoided and/or perturbations due to unknown masses being held in the hand or to external forces. Better understanding the movement parameters relevant for successfully producing these three-dimensional reaches would provide insight into natural neural control mechanisms, as well as suggest artificial control methods for rehabilitation (e.g., prosthetic limbs, functional electrical stimulation) and robotic systems.

Human reaching movements have symmetric bell-shaped velocity profiles when no accuracy constraints are present [1], but tend to become asymmetric as the required accuracy at the target increases [2]. This phenomenon may be a result of feedback processes [3], [4] responding to variability caused by noise in the nervous system [5].

In order to reach a target, the nervous system must perceive the states of the hand and target, plan motor commands based on these perceived states, and execute the commands using the musculoskeletal system. Variability during these three phases [6]–[10] all cause variability in the final movement. The motor system may plan and execute movements such that the cost of errors due to movement variability is minimized in the movement dimensions where the task imposes constraints [11], [12].

As a movement progresses, error detected by sensory feedback in conjunction with internal models of afference and efference is evaluated, and the remaining portion of the trajectory is modified accordingly [3]. These trajectory modifications may occur at discrete points in time, or may occur as a continuous process [13]. Several models hypothesize that reaching is composed of a series of submovements, where each submovement represents a discrete modification to the overall trajectory [3], [4], [14]–[21]. Neural correlates of submovements have been found [17], [22]–[24], lending support to this hypothesis. In addition, overlapping submovements have been used to describe human arm movements in able-bodied adults [25], [26], infants [27], and in adults recovering from stroke [28], [29]. Overlapping submovements have also been used to describe handwriting [30], wrist movements [16], and head movements [31].

Our motivation was to develop a model of human reaching sufficiently realistic to allow for corrections during the movement. Such a model could be used to simulate reaching trajectories and aid in development of future arm prosthetic devices. We have assumed that the current movement state is evaluated continuously [32] and that appropriate trajectory modifications are initiated at discrete points in time as overlapping submovements that add to the overall reaching movement.

In the first part of this study, we determined whether the submovement initiation process that would be necessary for a discrete-submovement model of human reaching could be captured and mimicked using deterministic Artificial Neural Networks (ANNs) that extract relevant parameters from ongoing movement kinematics. In the second part of this study, we determined whether these ANNs could together form a submovement-based “closed-loop” model (i.e., one that includes ongoing corrections for movement errors) that accurately predicts experimentally recorded arm reaching trajectories made to arbitrary targets.

To accomplish this, we decomposed experimentally recorded reaching movements into their submovement components using an optimization procedure, assuming that each submovement was a minimum-jerk trajectory [1], [25], [26], [33]. Then, separate ANNs were trained to learn the relevant parameters of the decomposed submovements: (1) initiation times, (2) durations, and (3) amplitudes. Finally, the ANNs were combined to form a closed-loop model that generated accurate reaches with trajectories similar to experimentally recorded trajectories. An early version of this work has been presented at a conference [34].

## Methods

### A. Ethics Statement

This experiment was approved by the MetroHealth System Institutional Review Board with protocol number IRB10-00126. Five able-bodied right-handed adult human participants were enrolled in this study. None of these participants were from vulnerable populations. Capacity to consent was determined over the course of multiple discussions with each potential participant, who verbally indicated that they understood the study and expressed interest in enrolling. Then, the participants provided written informed consent. This process was consistent with requirements DHHS 45 CFR Part 46, FDA 21 CFR Parts 50 and 56, and HIPAA 45 CFR Part 164.

### B. Experimental Setup

**Figure 1:**
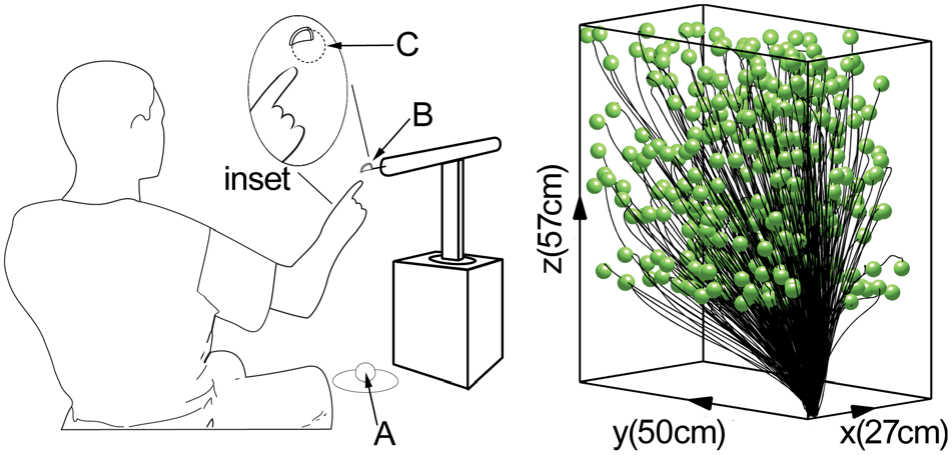
Experimental setup (left) and recorded position trajectories (right) for one participant. Participants made reaching movements from the start position (A) to a target presented on the end of a robotic actuator (B). The start position was a holographic sphere in an arm-rest-like position. The target position was displayed to the participants as a hollow sphere octant, one eighth of a sphere. From the displayed portion of the sphere, participants were required to imagine the rest of the sphere (inset, C). The recorded position trajectories (black traces) to each target (green spheres) were measured using an optical tracking system. The reaches shown in the figure were recorded from participant D.

Each of the five participants (labeled A, B, C, D, and E) made right-hand reaching movements from a starting arm position to a series of targets randomly positioned in the 3D reachable workspace in front of them (Fig. 1). Participants wore a rigid index-finger brace that completely immobilized the interphalangeal joints and partially immobilized the metacarpalphalangeal joint. The starting position mimicked the posture of the arm when resting on the armrest of a chair or wheelchair, and was cued using a sphere displayed holographically via two concave mirrors (Mirage, Opti-Gone International, Ojai, CA USA).

The target cue was a hollow sphere octant with an inside radius of 1.59 cm attached to a robotic actuator (HapticMaster, Moog in The Netherlands, Nieuw-Vennep, The Netherlands) with the concave side facing the participants. In spherical coordinates, the target cue encompassed a range of 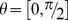 and 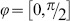, and allowed participants to estimate the center of the sphere as the intersection of the three orthogonal planes that “sliced” the sphere. This physical sphere octant provided enough visual cues for the participants to imagine the rest of the sphere. In software, a virtual spherical target of radius 1.27 cm was co-aligned with the center of the physical sphere octant. This arrangement enabled (and required) participants to move to the target without physically touching it. Performance in the task was judged relative to the co-aligned virtual target.

Three-dimensional fingertip and target positions were sampled at 100 Hz using an optical tracking system (Optotrak 3020, Northern Digital Inc., Waterloo, Ontario, Canada), with infrared markers placed at the fingertip and on the robotic actuator. Throughout the experiment, custom Simulink (Mathworks Inc., Natick, MA USA) software (1) recorded fingertip and target position data in real time from the Optotrak and (2) controlled the positioning of the robotic actuator.

The robotic actuator positioned the target at a series of random locations in the reachable workspace. When the actuator stopped for each target, an audible cue instructed the participant to initiate movement from the starting position to this target. Whenever the fingertip entered the virtual target, another audible tone sounded. Following a one second dwell period, an audible cue then signaled the participant to return to the starting position. The participant dwelled again for one second at the start position. Then, the robotic arm repositioned the target, which took approximately 3.5±0.25 seconds (mean and standard deviation). Then, an audible cue signaled the next reach. Participants were instructed to reach as quickly and as accurately as possible. Each participant made 375 reaching movements.

Post-processing was done offline in MATLAB (Mathworks Inc., Natick, MA, USA). Trials where participants did not appropriately follow the audio cues or physically touched the experimental apparatus, and trials where markers were occluded for more than five consecutive time steps (i.e., for >50 ms), were dropped from further analysis. After this process, participant datasets included 258, 332, 321, 351, and 353 reaches, respectively. The remaining data were digitally resampled to provide uniform time steps and to interpolate for occlusions less than 5 time steps, and smoothed with a zero-phase low-pass digital filter at 10 Hz to remove any artifacts unrelated to the movements. These datasets are available for download in Data S1.

### C. Submovement Decomposition through Optimization

An optimization procedure [33], [35], [36] was performed to find the set of minimum-jerk submovements whose summation closely approximated the measured trajectories. Minimum-jerk trajectories [1] have the form:
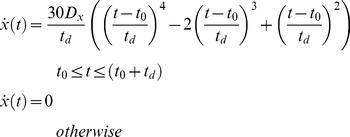
(1)where 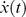 is the 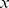 fingertip velocity, 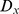 is the 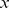 amplitude of the submovement, 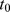 is the initiation time, and 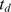 is the submovement duration. For this study, the minimum allowable value of 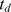 was set to 0.1 seconds, or the approximate duration of the force transient generated by a muscle twitch [37].

The summation of a discrete number of minimum-jerk submovements represents the reconstructed trajectory:
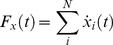
(2)where 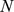 is the total number of submovements and 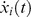 represents the 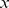 velocity of the 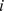-th submovement.

**Figure 2:**
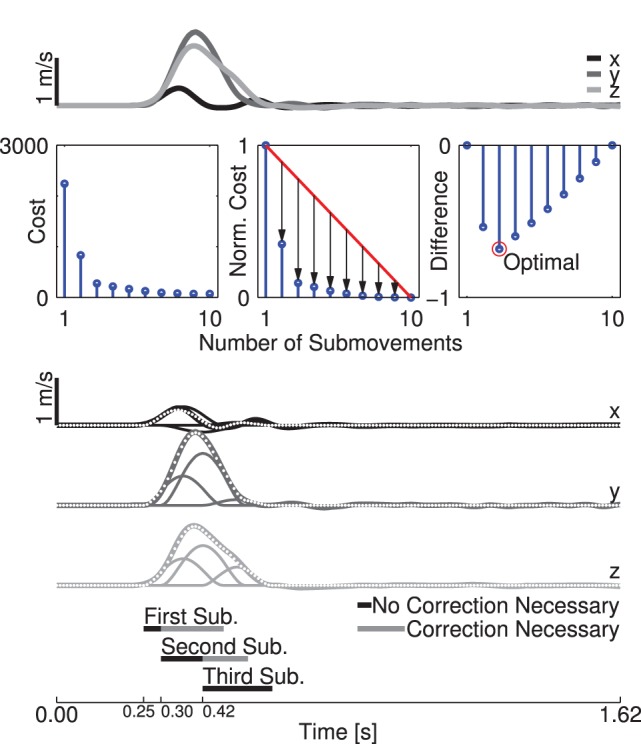
Submovement decomposition process. The top subpanel shows the x, y, and z velocity profiles of a recorded reaching movement. The second row of panels illustrates the results of the submovement decomposition process, including the optimization cost per number of submovements curve (left), the normalized version of this cost curve (middle), and the selection of the optimal number of submovements (right, see text for details). The next set of subpanels show, in each dimension, the measured trajectory (thick), the three optimal submovements (thin) that compose the measured trajectory, and the reconstructed trajectory that is the sum of the submovements (dotted white line). Finally, the timing of the three submovements is shown at the bottom. The dark lines represent the time when no correction is necessary, and the gray lines represent when a correction is necessary.

Submovements are three-dimensional, so similar expressions exist for 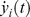 and 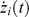. Therefore, each submovement can be completely described using five parameters: 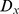, 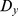, 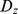, 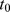, and 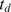. An optimization was performed to find the parameters that minimized the following cost function:
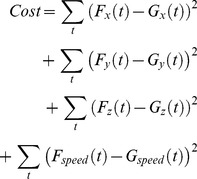
(3)where 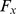, 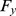, and 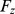 represent the 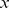, 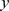, and 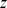 velocity components of the reconstructed trajectory, and 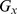, 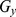, and 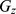 represent the 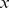, 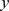, and 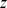 velocity components of the measured trajectory. The term that includes 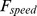 and 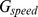 was introduced to prevent simultaneous submovements of opposite amplitudes from occurring [36]. These two parameters are defined as follows:
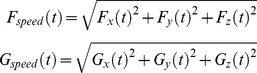
(4)Since the number of submovements 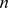 used in a particular reaching trajectory is unknown a priori, for each trajectory the decomposition was repeated with 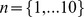, which encompasses the range of submovements decomposed in similar studies [29], [36], [38], [39]. As the number of submovements 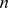 increases, the cost function 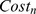 decreases but at a decreasing and, eventually, functionally insignificant rate. The optimal number of submovements 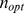 was determined using an algorithm that detects the point of maximum curvature in the optimization cost-per-submovement curve [40], selecting the minimum number of submovements required for near-asymptotic performance in predicting the actual movement:



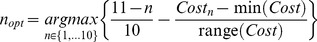
(5)An example of this process is shown in Figure 2. The top panel is the velocity trace of a single reaching movement. The second row of panels shows the cost-per-submovement curve 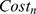 for this movement, which was normalized to range from 0 to 1. The point furthest from the diagonal line was picked as the optimal number of submovements (Eqn 5). Across all participants and all reaches, the decomposition process indicated between 2 and 5 submovements per reach, with averages of 2.94, 2.91, 2.79, 3.02, and 2.93 submovements per reach for the five participants.

### D. Submovement Initiation-Time Prediction

We trained the first of three ANNs to predict the initiation time 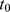 of each of the submovements, using kinematic and timing features that could be derived causally from the current and past movement states (see below) as the inputs [16]. The first submovement in each reach was assumed to begin at time 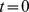. At each time step 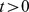 the network was trained to predict 1 or 0 corresponding to whether or not a subsequent submovement had begun, as indicated by the submovement decomposition process described above.

Table 1 summarizes the various movement features that were used to generate inputs to the ANN. These inputs were derived from the starting position, the target position, and the set of all submovements that had already been initiated prior to the current time step. Specifically, we used the Euclidean distance between every pairwise combination of positions listed in Table 1 under the Initiation Time column as inputs to this ANN. In addition to these inputs, several features related to movement timing were derived and used as inputs, as described in Table 1. These include an exponential transformation of time since the start of the submovement – a transformation that emphasized the early period of each submovement and improved prediction performance for corrections that occurred early in the submovement. Two binary inputs were also included, corresponding to whether the fingertip is (or will be) inside the target sphere.

**Table 1:** Inputs for Submovement Parameter Prediction.

Category	Input Name	Initiation Time	Amplitude	Duration
**Position Inputs** *	Position at start of reaching movement	1D	3D†	1D
	Position of target	1D	3D†	1D
	Current fingertip position	1D		
	Current submovement start position	1D	3D†	1D
	Current submovement end position	1D		1D
	Position at end of all initiated submovements	1D	3D†	1D
**Other Inputs**	Fingertip currently in target?	1 or 0		
	Fingertip in target at end of all initiated submovements?	1 or 0		
	Remaining time in current submovement	1D		
	Remaining time in current submovement, normalized‡	1D		
	Time since start of current submovement	1D		
	Time since start of current submovement transformed§	1D		
	Current fingertip velocity		3D1/4	1D1/4
	Current fingertip acceleration		3D1/4	1D1/4

*Position inputs underwent an additional processing step, taking the pairwise differences of the indicated position inputs. For 1D position inputs, differences were calculated as Euclidean distance. For 3D, the differences were calculated in three dimensions.

† Each 3D difference was normalized by the Distance To Target accounted for by All Prior Submovements (see text).

‡ Remaining time was normalized to the duration of the submovement.

§ The transformed t was e^−20t^.

1/4 Velocity and acceleration were transformed by taking the 4^th^ root.

These processed inputs were then used to train a feed-forward ANN with a five neuron hidden layer, with tansig (tangent-sigmoid) transfer functions. We found that this architecture provided a compromise between generalizability and prediction performance. With fewer than five hidden layer neurons, the network was unable to learn the input-output relationship, while performance did not dramatically improve with more than 5 neurons. We performed a 10-fold cross-validation with data assigned 80∶10∶10 into training, validation, and testing sets. Training data was used to compute the ANN parameters, validation data was used periodically during the training procedure to insure generality and to prevent over-fitting, and the testing data was used for the final assessment of predictive performance once the ANN was trained. The initiation time of each submovement was determined by running the ANN at each timestep of the immediately preceding submovement (of the same reach) until the ANN output first exceeded a threshold of 0.5. This timestep was then designated as the initiation time 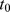 for the current submovement.

### E. Submovement Amplitude Prediction

We trained the second of three ANNs to predict the amplitudes 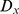, 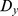, and 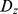 of each submovement of each reach. The inputs to this ANN were derived from the same set of features (Table 1) described previously. Position features were converted to relative distances by taking the pairwise differences in three dimensions. The magnitudes of the decomposed submovement amplitudes ranged from 0.46 cm to 56.24 cm. In order to prevent submovements with large amplitudes from inappropriately biasing ANN training, we applied an additional normalizing transformation. Specifically, we divided the position input features and output amplitudes by the “Distance To Target accounted for by All Prior Submovements (DTTAPS).” For the 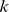-th submovement of a reach, it is defined as:
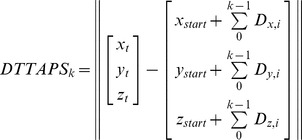
(6)where 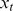, 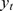, and 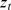 are the target position, and 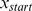, 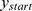, and 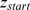 are the start position. For the first submovement of a reach there were no prior submovements, so the DTTAPS was simply the distance from start position to the target.

The instantaneous velocity and acceleration of the fingertip were also used as inputs to the amplitude prediction ANN because they provide information about the state of the current movement plan, the sum of the already-initiated submovements. We found that the velocity and acceleration distributions ranged over several orders of magnitude and were skewed with many small values and a few very large values. We transformed the velocity and acceleration by taking the 4^th^ root, a power transformation that simultaneously reduced dynamic range and skewness. These prevent large-valued velocities or accelerations from biasing the ANN training.

These inputs were used to train a feed-forward ANN with one hidden layer containing 10 neurons with tansig transfer functions, and three output neurons with logsig (log-sigmoid) transfer functions. Again, we found that this network configuration was a good compromise between generalizability and performance. The amplitude ANN was trained and tested using the same cross-validation used for initiation-time.

### F. Duration Prediction

We trained a third ANN to predict the duration 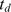 of each submovement from inputs derived from the same set of features (Table 1). As before, absolute positions were made relative by taking the Euclidean distance between each pair of positions. Velocity and acceleration inputs were transformed using the 4^th^ root as previously described. However, because duration is a scalar value, instead of using the three-dimensional transformed velocity and acceleration as before, we used the respective scalar magnitudes. These processed inputs were used to train a feed-forward ANN with one hidden layer containing 10 tansig neurons, and one logsig output neuron. Again, this network was found to be a good compromise between generalizability and performance. The ANN was trained and tested using the same cross-validation as before.

### G. Closed-loop Model for Simulated Trajectories

**Figure 3:**
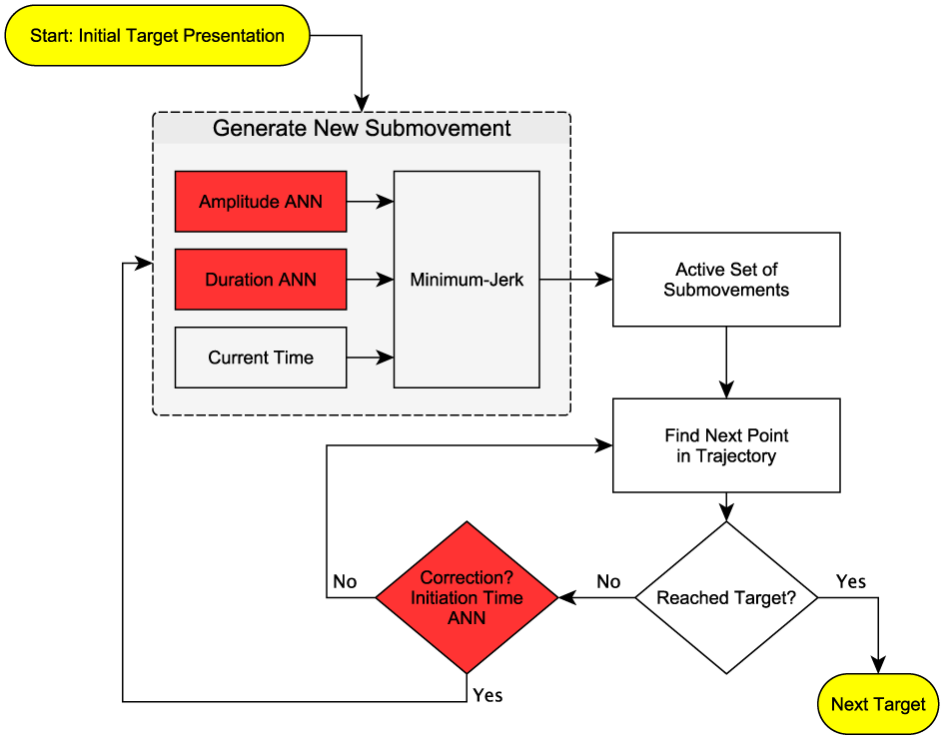
Schematic representation of the closed-loop model for trajectory prediction. The initial submovement of each reach is automatically triggered at time t = 0, when the target is presented. The initiation time, amplitude, and duration of each submovement are predicted using the respective Artificial Neural Networks (ANNs), and the resulting minimum jerk submovement is added to the set of active submovements until the target is reached.

The three separate ANNs were incorporated into a single closed-loop model (Fig. 3) of human arm reaching trajectory generation. In this closed-loop configuration, rather than using inputs derived from decomposition, the ANNs used inputs derived from the trajectory generated by earlier ANN predictions. This is a causal process that allows prediction errors to propagate to subsequent ANN inputs, as they do in actual reaching movements.

The initial submovement of a target-oriented reach began at time 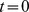. The amplitude and duration of this initial submovement were predicted by the corresponding ANNs. With these parameters, the first minimum-jerk submovement was initiated. At subsequent 0.01 s timesteps, the initiation-time ANN determined whether a corrective submovement should be initiated. If an additional submovement was necessary, its amplitude and duration were determined using the respective ANNs and the input data available at that time (i.e., the process was causal). The predicted trajectory was the sum of the minimum-jerk submovements parameterized by the corresponding ANN predictions.

This process continued until the simulated reach succeeded (hit, with one second dwell within a 1.27 cm target radius) or failed (missed, due to timeout conditions of three seconds of simulated time or 30 triggered submovements). The ratio of successes over attempts was the Target Acquisition Rate (Table 2). The performance of the closed-loop model was evaluated on each target from the cross-validation testing sets.

**Table 2:** Performance.

Category	Description	A	B	C	D	E
**Initiation** **Time ANN**	2^nd^ Submovement VAF	67.10%	65.70%	62.27%	59.70%	55.59%
	3^rd^ Submovement VAF	84.45%	72.03%	78.18%	75.83%	87.55%
	Overall VAF*	80.54%	70.07%	77.60%	72.76%	82.85%
	Sensitivity	98.36%	99.36%	98.06%	98.00%	98.65%
	Specificity	94.44%	97.40%	95.15%	98.06%	98.37%
**Duration ANN**	1^st^ Submovement VAF	51.97%	37.99%	40.66%	54.81%	37.85%
	2^nd^ Submovement VAF	59.73%	40.74%	54.60%	63.41%	60.20%
	3^rd^ Submovement VAF	61.79%	65.97%	39.59%	49.40%	46.79%
	Overall Prediction VAF	58.72%	50.96%	47.92%	60.19%	48.01%
**Amplitude ANN**	1^st^ Submovement VAF	22.48%	34.41%	30.96%	29.26%	30.46%
	2^nd^ Submovement VAF	84.11%	79.93%	86.09%	72.71%	72.46%
	3^rd^ Submovement VAF	94.78%	89.79%	94.94%	93.75%	95.08%
	Overall Prediction VAF	74.20%	67.64%	74.54%	67.32%	70.47%
**Closed-Loop** **Model**	Target Acquisition Rate	91.47%	100.0%	91.28%	99.43%	96.88%
	Dist. at Trajectory End†	0.9±0.39 cm	0.6±0.27 cm	0.8±0.39 cm	0.7±0.31 cm	0.7±0.34 cm
	Dist. at Failed Trajectory End†	1.6±0.68 cm	N/A	1.6±0.49 cm	1.3±0.80 cm	1.6±0.54 cm
	RMSE vs Actual Trajectory	3.01 cm	3.09 cm	4.32 cm	3.23 cm	3.21 cm
	VAF vs Actual Trajectory	97.94%	97.82%	95.95%	97.65%	97.50%

*Does not include first submovement that starts at t = 0 by definition. The Initiation-Time VAF calculations only include True Positives.

† Distance to center of target at end of predicted trajectory. Mean and standard deviation reported.

### H. Performance metrics

Each ANN was evaluated by comparing the ANN-predicted submovement parameters (i.e., initiation time, amplitude, and duration) to the corresponding parameters decomposed directly from experimentally recorded trajectories. In addition, the performance of the closed-loop model was evaluated by comparing generated trajectories to the experimentally recorded trajectories.

We used the metrics variance-accounted-for (VAF) and root-mean-squared error (RMSE) to quantify the comparison. The following formulation of VAF was used:
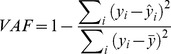
(7)where 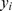 is the 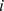-th observed value (either directly observed or decomposed from observed), 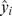 is the corresponding predicted value, and 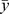 is the mean of the observed values.

## Results

### A. Predictions of movement parameters

**Figure 4:**
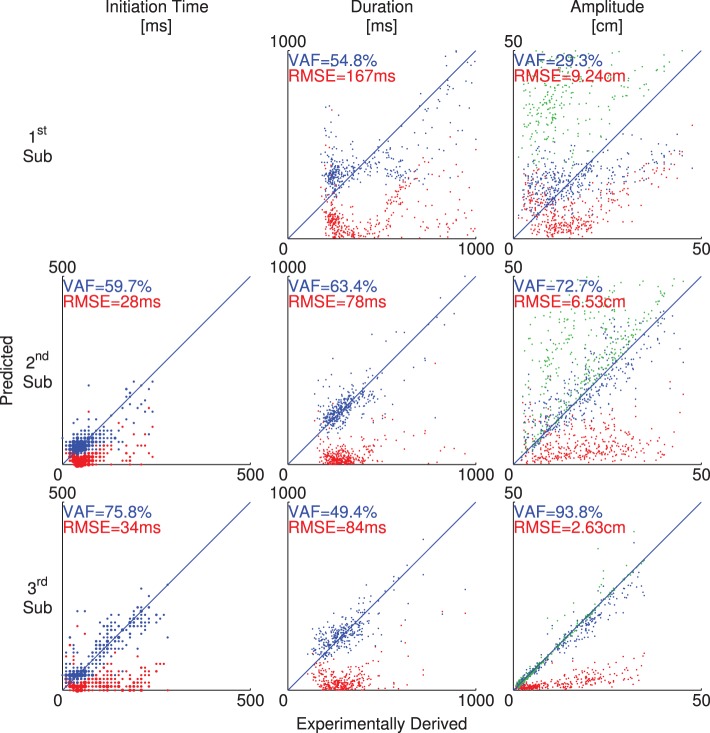
Prediction performance of initiation-time, duration, and amplitude ANNs for participant D. The columns of subpanels correspond to the initiation time, duration, and amplitude predictions, respectively. The rows of subpanels correspond to predictions for the first, second, and third submovements of each reach. Within each panel, the blue points indicate the relationship between the experimentally derived value of a submovement parameter (x axis) and its ANN-predicted counterpart (y axis). The red points indicate the error in the prediction. For amplitude, the plotted results represent overall 3D magnitudes and errors, not errors in single dimensions. The green points in the amplitude column indicate the remaining distance to target when the submovement was initiated. The y values indicate the remaining distance, and the x values are the corresponding amplitude of the submovement that was made at that time. Also indicated in each subpanel are the average %VAF and RMSE for this participant.

Figure 4 illustrates the performance of the three ANNs in predicting submovement initiation time (leftmost column), submovement duration (middle column), and submovement amplitude (rightmost column) for participant D. The top row of panels represents the first submovement, the middle row represents the second submovement, and the bottom row the third submovement. In each panel, the blue points plot the ANN predicted quantity (vertical axes) versus the corresponding experimentally derived quantity (horizontal axes). Perfect prediction would be represented by all blue points falling on a line of slope 1.0 (included for reference in each subpanel). The red points indicate the magnitude of the prediction error, equivalent to the vertical distance from each blue point to the diagonal line. The %VAF and RSME for the prediction of the various parameters are indicated for this participant in each panel. Note that the parameters for submovements four and above are not shown in Figure 4 because few movements contained more than three submovements.

Table 2 summarizes several performance metrics for offline and closed-loop mode, for all participants. Figure 4 and Table 2 will be used below to illustrate the characteristics of one participant in detail and a summary of properties across all subjects, respectively.

#### 1) Initiation-Time

The leftmost column of panels in Figure 4 illustrates the ability of the ANN to predict submovement initiation time. In this figure, initiation times are expressed relative to the start of the previous submovement. The initiation time of the first submovement was 0 for all movements by definition, thus the blank panel on the top left. The accuracy of the predictions of initiation time for the 2^nd^ and 3^rd^ submovements is indicated by distance of the blue points from the slope = 1.0 line. Errors in the predicted initiation times were clearly smaller than the magnitude of the initiation times (the blue points are larger than the red points). Across all subjects (including the subject illustrated in Figure 4), the VAF for initiation time predictions increased for the 3^rd^ submovement relative to 2^nd^ (compare the first two lines in Table 2), and the overall VAF was at least 70% (Table 2, third line). These VAF values were calculated by lumping together predictions from all cross-validation folds.

The performance measures associated with Figure 4 are only meaningful for True-Positive (TP) cases when the model predicted a correction when there was a correction according to the experimentally based submovement decomposition. False-Positives (FP, i.e., when the Initiation Time ANN predicted a correction when a correction was unnecessary, according to decomposition), False-Negatives (FN, i.e., when this ANN did not predict a correction that was actually necessary according to decomposition), and True-Negatives (TN, i.e., when this ANN did not predict a correction when one was not necessary according to decomposition) cannot be represented using VAF and RMSE. In these cases, the performance of the model was quantified using sensitivity and specificity.

*Sensitivity* is defined 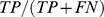 and is the percentage of experimentally decomposed submovements that needed corrections that were correctly predicted by the initiation-time ANN. Sensitivity across participants for predicting corrections was at least 98% (Table 2). These are the submovements represented in the leftmost column of Figure 4. This sensitivity suggests that for up to 2% of the submovements, the ANN did not make a correction when it should have.

*Specificity* is defined 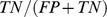 and represents the percentage of submovements that did not need corrections that were correctly recognized as such by the initiation-time ANN. The specificity was greater than 94% across all participant datasets (Table 2), suggesting that for less than 6% of the submovements, the ANN triggered corrections when it should not have.

#### 2) Duration

The second column of panels in Figure 4 illustrates the predictive performance of the duration ANN. The format and color-coding of these panels are the same as for the Initiation Time predictions in the left column. As can be seen, the duration predictions tended to fall close to the diagonal line. For this particular participant, the errors in duration predictions were less than the durations themselves, except for the shortest durations where some red points exceed the diagonal. For longer durations, the error increased but was less than the total duration. For every participant, the %VAF for the 1^st^ submovement duration prediction was lower than the overall %VAF (Table 2). The overall VAF ranged from 47.9% to 60.2% (Table 2, Duration section, fourth line). As with initiation-time, these values were calculated by lumping together predictions from all cross-validation folds.

#### 3) Amplitude

The rightmost column of Figure 4 illustrates the predictive performance of the amplitude ANN for the same participant and for the first three submovements. The format and color-coding of these panels are similar to the Initiation Time predictions in the left column, with three exceptions. First, the blue points represent the magnitude of the predicted amplitude vectors (vertical axis) versus the magnitude of the corresponding experimentally derived amplitude vectors (horizontal axis). This is because the amplitude ANN predicted three dimensional amplitude vectors, while the other ANNs predicted scalar values. Second, the prediction errors indicated by the red points correspond to the Euclidean distance between the predicted and experimentally derived amplitude vectors. Note that perfect prediction of amplitude magnitude does not imply perfect prediction of amplitude vector direction. The %VAF and the RMS errors, calculated based on the 3D vector amplitudes, are indicated in each panel. Third, the green points in each amplitude subpanel indicate additional information, the distance to target at the moment of submovement initiation (vertical axis) versus the magnitude of the experimentally derived submovement amplitude (horizontal axis). This relationship is an indication of whether or not submovements cover the remaining distance to target. If the green points fall on the diagonal line, then the magnitude of the experimentally derived submovement amplitudes match the remaining distance to target.

As can be seen from blue points in Figure 4, amplitude predictions became progressively more accurate (i.e., bunched closer to the diagonal line) as the reach progressed through successive submovements. The RMS error decreased with increasing submovement index as well. The 1^st^ submovement amplitude predictions had much lower %VAF than subsequent predictions. These trends were true across all participants (Table 2). As before, %VAF was calculated by lumping together predictions from all cross-validation folds.

The magnitude error, indicated by the vertical distance from blue points to the diagonal line, is correlated with the Euclidean error, indicated by the red points. This is especially noticeable in the first submovement (Figure 4 top subpanel). These two errors would be identical if the direction were predicted perfectly. For this participant, for all submovements, 89.1% of the differences in errors were less than 2 cm, and 97.1% were less than 4 cm. These are small compared to the range of submovement magnitudes in Figure 4 and indicate that most of the prediction error can be explained by magnitude error rather than direction error.

For the first submovement, very few of the experimentally derived amplitudes covered the remaining distance to target, as the green points fell above the diagonal line. This indicates that the decomposed initial submovements tended to undershoot the actual distance-to-target. The blue points were much closer to the diagonal line than the green points were. This was also true for the second submovement.

For the third submovement, many of the experimentally derived amplitudes covered the remaining distance to target, as the green points fell very close to the diagonal line. This is consistent with the fact that, for many of the reaches, the decomposition process produced three submovements and the third submovement reaches the target.

### B. Closed-loop Model for Simulated Trajectories

**Figure 5:**
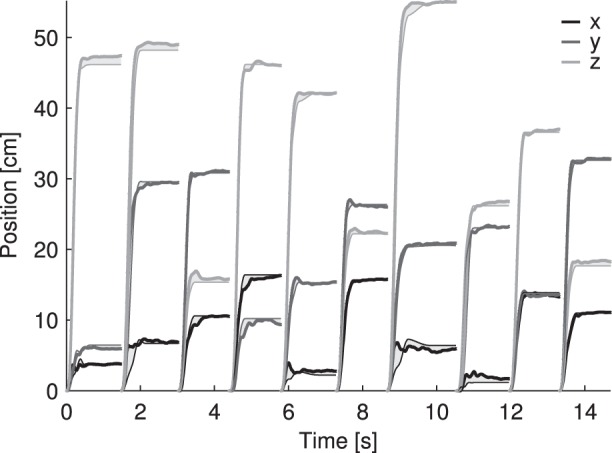
Example closed-loop simulated trajectories. Selected position trajectories from participant D are shown. Thick lines represent measured trajectories and the thin lines represent simulated trajectories. Error between the trajectories is shaded in light gray.

The three individual ANNs were combined into a closed-loop model (Fig. 3) that predicted overall movement trajectories (Fig. 5) based on previous movement characteristics. Figure 5 gives an example of the trajectories predicted by the closed-loop model for 10 reach targets selected from participant D’s dataset. The simulated 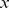, 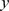, and 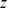 position trajectories are shown as thin lines. For comparison, the measured positions for the same reaches are indicated in thick lines.

While there are minor differences between the experimentally recorded and the simulated trajectories, the errors are very small relative to the total distance from the starting location to the targets. The errors typically manifest as overshoots or undershoots in one or more dimensions. For the trajectories shown, the VAF between the actual and predicted trajectories across the entire movement was 98.48% and the RMS error was 2.47 cm. Across participants, the overall VAFs of closed-loop model predicted trajectories were quite high and very consistent (95.9%–97.9%), and the RMS error was small and consistent (3.01–4.32 cm) (Table 2, Closed-Loop).

Over all participants, the target acquisition rate of the predicted trajectories ranged from 91% to 100%. The mean distance to the centers of the targets at the end of predicted trajectories was less than 1 cm, and the mean distance for missed targets was at most 1.6 cm. For comparison, the radius of the target sphere was 1.27 cm, and the distance from start position to target was 43.5±11.2 cm (mean and standard deviation).

**Figure 6:**
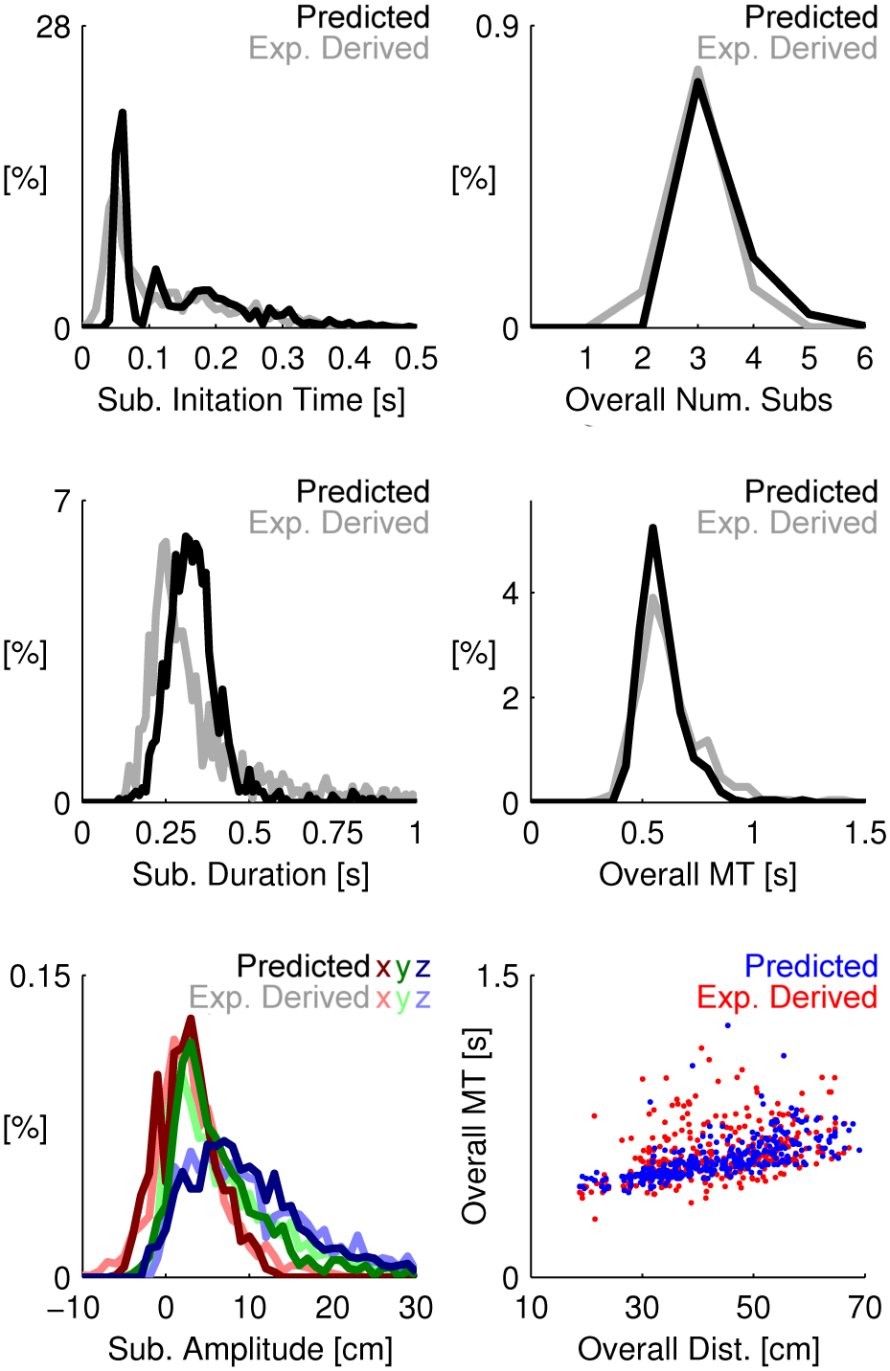
Closed-loop movement and submovement parameters, predicted versus experimentally derived. The three left panels show the distributions of initiation times (top left), durations (middle left), and amplitudes (bottom left) for participant D. Within each panel, the dark lines represent the predicted distributions, and the light lines represent the corresponding experimentally derived distributions. Initiation times are shown relative to overall movement start. The three right panels show the respective distributions for the number of submovements (top right), the overall movement time (MT) (middle right), and the MT versus Distance relationship (bottom right). For MT vs Distance, blue points indicate ANN predictions and red points indicate experimentally recorded values.

To evaluate the effect of ANN prediction error propagation in closed-loop, the predicted parameter distributions were compared to the experimentally derived distributions across all reaches (Fig. 6). The three panels on the left represent the distributions of submovement initiation time (shown relative to overall movement start), duration, and amplitude for participant D. The three panels on the right represent the distributions of overall number of submovements, Movement Time (MT), and a plot of MT versus Distance to Target. For the top four panels, the dark lines represent the predicted distributions, and the light lines represent the experimentally derived distributions. In the submovement amplitude panel (bottom left), the 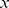, 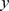, and 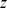 components are shown separately in red, green, and blue, respectively. For perfect predictions, the predicted distributions should match the experimentally derived distributions. For the MT vs Distance (bottom right) panel, blue points represent the predicted data, and red points represent the experimentally derived data. For perfect predictions, the blue points should fall in approximately the same regions as the red points.

Qualitatively, the initiation time, amplitude, submovement number, and overall MT distributions closely matched their experimentally derived counterparts. Duration predictions were also close although there was a 0.06 s horizontal shift between the peaks of the two distributions (Fig. 6). When the first submovement of each reach was not included in the analysis, the peak shift reduced to 0.02 s.

The predicted MT to Distance relationship is less variable than the experimentally derived MT to Distance relationship. For this figure the experimental MT was defined to start at the beginning of the first decomposed submovement, and does not include the one second dwell period. Both the experimentally derived and predicted MTs increased as overall distance increased.

**Figure 7:**
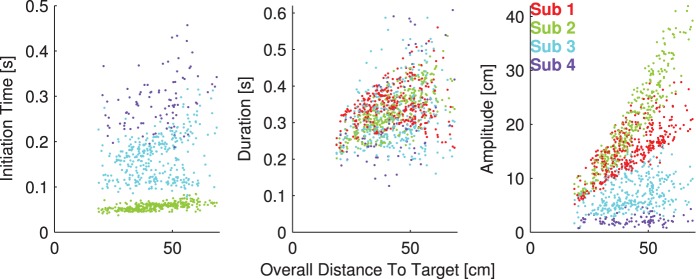
Closed-loop submovement parameter predictions, plotted versus overall distance to target. The three panels represent the initiation time (left), duration (middle), and amplitudes (right) of the submovements, respectively. The four colors represent the first four submovements, labeled according to the legend in the rightmost panel. By definition, the first submovements initiated at time 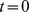, so these points were omitted. Subsequent initiation times are shown relative to overall movement start. A small amount of jitter has been added to the initiation times to facilitate visualization. The amplitude points plotted are the scalar magnitudes of the submovement amplitudes.

We also investigated the relationship between the predicted submovement parameters and the overall distance to target (Fig. 7), one of the inputs common to all three ANNs (Table 1). The three panels of this figure correspond to the predicted initiation times (shown relative to overall movement start), durations, and amplitudes, respectively, plotted against the distance to target. A small amount of jitter (<5 ms) was added to the initiation times to facilitate visualization. The colors of each point correspond to which submovement it represents, with red corresponding to the first submovement, green to the second, and so on (see legend in right panel). Note that only the first four submovements are shown. The initiation times and amplitudes were strongly related to submovement number, but durations were not. Many of the second submovements began at around 50 ms after movement initiation. In addition, some predictions were strongly correlated to the distance to target. The four strongest correlations were in the second submovement initiation time (linear regression R^2^ of 45%), second submovement duration (R^2^ of 49%), and first and second submovement amplitudes (R^2^ of 56% and 88%, respectively). Many of the predictions were not correlated to distance to target, such as the first submovement duration (red points, middle panel) or the third submovement initiation time (light blue points, left panel).

## Discussion

We have found that three Artificial Neural Networks (ANNs) can accurately predict the essential parameters (initiation time, amplitude, and duration) of submovements that compose human reaching movements, using continuous kinematic information derived from preceding submovements. We have further demonstrated these three ANNs can be combined into a closed-loop model that sums predicted submovements into trajectories that have similar shape, accuracy, and submovement parameters as actual trajectories. This ANN-based model can thus be used to simulate human reaching trajectories with error corrections. As such, it could be used to simulate the command interface for various rehabilitation interventions such as Brain-Computer Interfaces (BCIs).

### A. Decomposition of 3D Human Reaching Movements into Submovements

The set of rapid and accurate radial (proximal to distal) reaching movements we recorded (Figure 1) is a common first component of many activities of daily living relevant for rehabilitation of individuals with arm paralysis [41]. These movements do not represent all potential directions or speeds of reaching movements, but they do represent the most important movements for upper-extremity prostheses. Our task was performed in 3D, against gravity, and to non-repeated targets, with a large range of distances to target (20 to 70 cm, Figure 6).

In order to train the ANNs, we needed to know the start times, durations, and amplitudes of submovements that occur during these 3D arm reaches. This information was determined by decomposing experimentally measured reaching movements into their constituent submovements. One common decomposition method involves parsing movements at landmarks such as zero-crossings in the velocity, acceleration, or jerk traces of the recorded trajectory [16], [19], [21]. The second method relies on fitting a number of overlapping basis function curves to the movement. An advantage of the basis function method is that, by definition, each submovement it decomposes has a consistent parameterization that we leveraged to make trajectory predictions. The implementations of the basis function method differ in terms of whether the trajectory is represented in terms of Cartesian [26], [33], tangential [39], or joint angle coordinates [38]. Rohrer & Hogan [33] proposed 3D Cartesian velocity decomposition into minimum-jerk submovements, but did not actually implement this. To our knowledge, our study is the first implementation of 3D submovement decomposition from Cartesian velocity trajectories.

While there are an infinite number of basis functions that could have represented the reaching trajectories, velocity profiles of rapid aimed movements are approximately bell-shaped [1] and evidence from stroke recovery and developing infants suggest that movements are composed of separate bell-shaped submovements [27]–[29]. We used the minimum-jerk trajectory because it has been shown to represent reaching trajectories [1] and it has a low number of parameters per submovement (start time, duration, and 3D amplitude) with defined start and end times that reduce its computational complexity relative to other basis functions such as the lognormal support-bounded or Gaussian functions [42].

Rohrer and Hogan [33] showed that many characteristics of submovements were consistent regardless of whether minimum-jerk, Gaussian, or lognormal support-bounded basis functions were used. This suggests the ANNs would have successfully learned the relationships between the decomposed submovements even if we had used a different basis function.

### B. Prediction Accuracy

The predictions of the overall movement trajectories by the combined three-ANN Closed-Loop Model were extremely accurate, with high %VAF and low RMSE (Fig. 5, Table 2). Almost all the targets were reached, and on missed reaches, the mean distance to target centers (Table 2) was still only slightly larger than the 1.27 cm target radius. The functional consequence of such misses, for many real-world reaching tasks, would typically be insignificant.

In addition, the predictions of the submovement parameters were also quite good, especially for the initiation time and amplitude (Fig. 4, Table 2, Fig. 6). Furthermore, the rates for incorrectly predicting a submovement that did not actually occur and NOT predicting one that did occur were very low (Table 2), and led to realistic submovement numbers in the closed-loop trajectory simulations (Fig. 6).

Interestingly, the predictions of the submovement parameters tended to improve with the submovement number, i.e. the model became more accurate as the hand approached the target. This could arise in part simply because of the mathematics. As a movement proceeds, subsequent submovements begin closer to the target so the range of possible relevant parameters progressively declines. However, the improvement in predictions with decreasing distance to the target may also reflect a movement strategy where more variation is allowed early in the movement, but as the arm approaches the target, the movement becomes more carefully controlled. This view is very similar to the “two-phase” strategy proposed by Rand & Shimansky [43]. As variation decreases, the submovement parameters become more predictable.

### C. Do submovements represent error corrections?

Human arm movements appear to (1) have early movement variation and (2) compensate for this variation by planning submovements that undershoot the target [44], [45]. The amount of undershoot is proportional to the amount of variation expected at the end of the initial submovement [45] such that even with the variation, target overshoots (and the associated energy-inefficient movement-direction-reversing error correction) rarely occur. This target undershoot was observed in the decomposed submovements and in the predicted submovement amplitudes (Fig. 4).

An important feature of this description is that the amount of variation expected can be learned over time [3] and allows for strategies to develop where corrections are not necessarily made in response to sensory feedback. In our results, the distribution of experimentally derived initiation times peaked at approximately 50 ms for the featured participant (Fig. 6). The ANN predictions also showed this pattern (Fig. 6 and 7) especially in the second submovement. It is possible that the early (∼50 ms) corrections of each reach were made as part of a feed-forward strategy developed in response to the early movement variation, while the later corrections were made with sensory feedback. However, it is also possible that the 50 ms peak is an artifact of our particular choice of basis function – a different submovement shape could potentially better fit the beginning of each reach and shift the initiation time peak to a higher time. These possibilities could be explored in a future study designed to systematically affect the amount of feed-forward control during reaches. For example, enforcing a short movement time requirement might encourage feed-forward behavior, while making the target invisible until movement start might discourage it. These, or related interventions, would provide context for the interpretation of time intervals between subsequent submovements.

Another question is whether corrections are implemented via submovements at all. Elliot and colleagues [46] suggested that corrections to reaching trajectories either occur as a series of overlapping submovements that appear continuous when summed together, or, that corrections are made continuously through graded adjustments of muscle gain. Because these strategies could result in similar continuous trajectory profiles, it is difficult to distinguish between them solely based on kinematics. Several groups have identified neural correlates of submovements in single-unit [17], BOLD [24], and EEG recordings [22]. It has also been shown that cortical neurons may represent movement fragments that add to form trajectories [47], a necessary feature of submovement-based motor control. Yet even with this evidence it is possible that the neural activity represents not submovements but instead continuous movement parameters that happen to be correlated to the onset of decomposed submovements.

Using a kinematics approach with non-overlapping submovements [19], Dounskaia and colleagues [48]–[50] suggested that rather than directly mediating accuracy at the target, most submovements were related to motion termination or were by-products of slow movement speeds. Motion termination submovements are potentially reconcilable with our view as they can also be considered gross error corrections. However, the view that submovements are by-products of slow movement speed suggests a more continuous non-submovement-based control strategy that is quite different from the various studies [14], [16], [27] on which we based our model. Further work will be needed to explore these hypotheses, especially in the context of overlapping submovements.

Our closed-loop model incorporates overlapping submovements that are assumed to represent distinct error corrections that the motor system purposefully but subconsciously initiates at discrete points in time [14]. The submovements could be initiated in response to information derived from visual or proprioceptive feedback, an internal model of expected afference and efference, task constraints, or other criteria that the motor system may optimize for [3], [17], such as energy expenditure [51], smoothness [1], or straightness [43], that could manifest as a feed-forward correction. We did not assume that error corrections were solely made in response to accuracy at the target.

Error corrections are difficult to model because their governing processes are not directly observable. In the current study, submovements decomposed from reaching trajectories provided a framework in which to observe the error correction process and allowed us to build a closed-loop trajectory prediction model. This approach does not depend on whether or not the motor system actually represents movements as submovements, and can be extended to different sets of causal movement-related data, or to different kinds of reaching movements. For instance, decomposed minimum-jerk submovements have been used to study nonconscious cognitive processes during reaching [35].

It was not a goal of the current study to prove or disprove the hypothesis that submovements represent error corrections. We assumed this model of error corrections based on literature, and used it to build a model capable of making error corrections during simulated arm reaches. Approaches that are not based on submovements, such as the two-phase strategy of Rand & Shimansky [43], [52], could also work and deserve further investigation.

### D. Limitations

We demonstrated that ANNs trained on kinematic information alone can accurately predict many aspects of reaching movements. However, because the ANNs were deterministic, the predicted trajectories did not exhibit the variation that is expected of repeated reaching to the same target. This is a result of not taking into account neuromotor noise [5] that causes variability, and limits the prediction %VAF that the deterministic ANNs can achieve. For example, for a particular measured reach, if the participant’s neuromotor noise state initially biased the reach in a certain direction, this would not be reflected in the corresponding kinematic ANN inputs. This is likely why prediction of the initial submovement amplitude and durations were worse than the overall prediction performance (Table 2 Duration and Amplitude, Fig. 4). The relatively poor initial duration predictions (Fig. 4) were largely responsible for the difference between closed-loop predicted and experimentally derived duration distributions (Fig. 6). These limitations could be addressed in a future study that incorporates neuromotor noise in the form of pre-movement neural activity [8] and including these in the ANN inputs.

The ANNs used in our model were black boxes rather than physiologically structured. By definition, the ANN outputs were systematically but nonlinearly related to the inputs, making it difficult to draw conclusions about the role of any particular input in making predictions. For instance, the overall distance to target appears in each set of ANN inputs (Table 1, the difference between Position at start of reaching movement and Position of target) but is only highly correlated to some predicted parameters (Fig. 7). Further analysis is necessary to determine the nature of the dependence of ANN predictions on the distance to target or other particular inputs.

We adopted the ANN structure because it is capable of learning nonlinear relationships, such as the submovement generation process, in a general way. Future work could examine the physiological mechanisms of such behavior and propose a more specific, mechanism-based substitute for the ANNs.

### E. Implications

Submovements decomposed from 3D Cartesian coordinates provide enough kinematic information to predict subsequent submovement parameters. While prediction quality would likely improve with richer inputs either in the form of higher-dimensionality kinematics (joint angles) or the addition of neural information, we found that 3D Cartesian kinematics are sufficient and are computationally simpler.

It has been shown that performance of potential rehabilitation interventions in open-loop (i.e., offline predictions without error correction) is often not a good predictor of closed-loop performance [53], [54]. The reason for the discrepancy is that a human user in closed-loop would be able to correct for errors. The ANN-based closed-loop model can be used to simulate human reaching that incorporates error corrections and would allow interventions that involve arm reaching, including BCIs, to be tested in simulated closed-loop situations without a human user, prior to being evaluated in vivo.

## Conclusion

Given a set of kinematic input variables, individual ANNs can predict the initiation times of subsequent submovements along with their amplitudes and durations. Also, these ANNs can form the basis of a closed-loop model that simulates trajectories that are similar to their experimentally measured counterparts.

This work is consistent with, but does not prove, the hypothesis that movements are composed of submovements that represent error corrections. Also, this study produced a practical submovement-based trajectory generator modeled after actual reaching movements that is capable of making error corrections. We anticipate that this type of model will be a useful development tool for the rehabilitation community.

## Supporting Information

Data S1 **This zip file contains five .csv files corresponding to the reaching kinematics recorded from each of the participants.** The .csv files are named according to participant, labeled A through E.
(ZIP)
